# Routine Ketorolac Use for Postoperative Pain Does Not Increase Bleeding Risk After Hysterectomy

**DOI:** 10.3390/jcm15020869

**Published:** 2026-01-21

**Authors:** Grace M. Pipes, Rebecca J. Schneyer, Kacey M. Hamilton, Ogechukwu Ezike, Katharine Ciesielski, Kelly N. Wright, Raanan Meyer, Matthew T. Siedhoff

**Affiliations:** 1 Department of Obstetrics and Gynecology, Cedars-Sinai Medical Center, Los Angeles, CA 90048, USA; grace.pipes@cshs.org; 2 Division of Minimally Invasive Gynecologic Surgery, Department of Obstetrics and Gynecology, Cedars Sinai Medical Center, Los Angeles, CA 90048, USA; 3 The Dr. Pinchas Bornstein Talpiot Medical Leadership Program, Sheba Medical Center, Tel Hashomer, Ramat-Gan 52621, Israel; 4 Faculty of Medicine, Tel-Aviv University, Tel-Aviv 69978, Israel

**Keywords:** bleeding complications, hysterectomy, ketorolac, Minimally Invasive Gynecologic Surgery, pain management, postoperative

## Abstract

**Background/Objective:** Ketorolac is an effective alternative and addition to opioids for postoperative pain control; however, there is concern of perioperative bleeding risk with its use. Within gynecology, this risk has not yet been explored in the context of hysterectomy. This study aimed to evaluate the risk of postoperative bleeding complications with ketorolac administration in the context of hysterectomy. **Methods:** This was a retrospective cohort study that included all patients who underwent hysterectomy for benign indications between 2015 and 2024 at a quaternary care academic hospital. Inclusion criteria were any type of hysterectomy during the study period, while exclusion criteria were malignancy and peripartum status. Complication data for up to thirty days post operation were collected. Multivariable regression analysis, including age, American Society of Anesthesiology category, use of celecoxib before surgery, anticoagulant treatment, uterus size, surgical approach, increased surgical complexity, and lysis of adhesions, was performed to identify the adjusted odds of postoperative bleeding complications. The primary outcome was a composite of any postoperative bleeding complications by use of postoperative ketorolac, including postoperative transfusion, readmission, or reoperation for bleeding. **Results:** In total, 4236 patients underwent hysterectomy for benign indications during our study period, of which 76% (*n* = 3236) received ketorolac postoperatively. The composite postoperative bleeding rate was lower in the ketorolac group (2.1% vs. 4.1%, *p* = 0.001). There was no association between ketorolac use and risk of postoperative bleeding in multivariable regression analysis (aOR 1.02, 95% CI 0.36–2.88). There was no difference in overall intraoperative or perioperative complications (*p* = 0.070 for both). Major perioperative complications were less likely in the ketorolac group (*p* = 0.046). Additionally, there were no differences in postoperative complications except for ileus, which was less likely in the ketorolac group (*p* = 0.034). **Conclusions:** Ketorolac administration was not associated with a higher risk of bleeding complications after hysterectomy, including when celecoxib was used preoperatively as part of an enhanced recovery protocol. It may safely be administered as an opioid-sparing pain medication in this setting.

## 1. Introduction

Ketorolac, similar to other non-steroidal anti-inflammatory drugs (NSAIDs), inhibits prostaglandin synthesis by inhibiting cyclooxygenase’s ability to convert arachidonic acid into prostaglandins, prostacyclin, and thromboxane [1]. Ketorolac is effective for the treatment of postoperative pain and reduces the need for opioid use [2,3].

Enhanced Recovery After Surgery (ERAS) is a well-established set of perioperative protocols that includes multimodal postoperative analgesia [4,5]. Multimodal analgesia reduces patient reliance on opioids and therefore their potential side effects—nausea, sedation, and fatigue—and the risk of addiction. Non-opioid alternatives include NSAIDs, acetaminophen, gabapentin, and regional anesthesia for first-line pain management. Ketorolac is the only parenteral NSAID available in the United States (U.S.) and is therefore an important component in ERAS protocols.

In the perioperative setting, there has been concern regarding ketorolac’s potential to impair platelet function and thus increase the risk of bleeding. Although it does significantly increase bleeding time, it does not significantly affect platelet aggregation and therefore may not have a clinically meaningful impact on postoperative bleeding [6,7].

The Federal Drug Administration (FDA) warns against perioperative use of ketorolac due to a post-marketing surveillance cohort study that found associations with the peri- and postoperative use of ketorolac and bleeding risk [8,9,10]. An older study reported a significant increase in operative site bleeding with ketorolac use, specifically in older patients, at higher dosages, and for courses lasting longer than five days [9]. However, recent meta-analyses evaluating NSAIDs—including ketorolac specifically—have contradicted these findings [11,12].

The risk of bleeding differs greatly between surgical procedures. Certain procedures, such as tonsillectomy, have demonstrated a trend towards increased hemorrhage with ketorolac use, leading to a warning against its use in these procedures [13,14]. Within gynecology, there are limited data surrounding the use of ketorolac perioperatively and none specific to hysterectomy, which is among the most common procedures for women in the US [15,16]. This study aims to evaluate the risk of postoperative bleeding complications with ketorolac administration in the setting of hysterectomy for benign indications.

## 2. Materials and Methods

### 2.1. Study Design and Data Source

This study was a retrospective cohort study that included all patients who underwent hysterectomy for benign indications between January 2015 and March 2024 within the Department of Obstetrics and Gynecology at a quaternary care academic hospital. The study received approval from the Cedars-Sinai Medical Center Institutional Review Board.

### 2.2. Inclusion and Exclusion Criteria

We included patients who underwent any type of hysterectomy performed for benign indications during the study period. Exclusion criteria were malignancy and peripartum hysterectomy. Cases with malignancy suspected preoperatively were excluded as well. Peripartum hysterectomies were excluded as they inherently have high bleeding risks and often require intraoperative blood transfusions.

### 2.3. Ketorolac Administration

At our institution, ketorolac is typically administered at the end of surgery, before anesthesia reversal. Administration is at the discretion of the provider. The usual dose is 30 mg given intravenously. Repeat doses of 15 to 30 mg are given as needed every 6–8 h during postoperative admission, and the maximal dose is 120 mg per day.

### 2.4. Patient Population and Data Elements

Data were collected from the electronic medical record. Baseline patient characteristics included age, body mass index (BMI), American Society of Anesthesiologists (ASA) classification, race, ethnicity, preoperative anemia, and insurance type. Operative characteristics included indications for hysterectomy, surgical approach (conventional laparoscopy, robotic-assisted laparoscopy, abdominal, and vaginal), hysterectomy type (total and supracervical), uterine size, mini-laparotomy usage, conversion to laparotomy, concomitant procedures, estimated blood loss (EBL), and same-day discharge. We further collected data on celecoxib administration, another type of NSAID routinely administered prior to surgery as part of ERAS protocol, and perioperative anticoagulant use.

### 2.5. Complication Definitions

The composite outcome for postoperative bleeding included any readmission for bleeding, postoperative transfusion, or reoperation for bleeding within 30 days. Perioperative complications included intraoperative and postoperative complications within the same period. Intraoperative complications included any of the following: bladder, bowel or ureteral injury; excessive intraoperative bleeding; blood transfusion; and other rare complications.

Intraoperative complications were classified using the Classification of Intraoperative Complications (CLASSIC) scale, and postoperative complications were classified using the Clavien–Dindo scale [17,18]. These scales are widely accepted, standardized methods for grading and categorizing surgical complications based on severity and the interventions required to manage them. Intra- and postoperative complications were further classified into minor and major. For both intra- and postoperative scales, grades 1–2 were defined as minor complications and grades 3–5 as major.

Postoperative complications included infections (pelvic abscess, superficial surgical site infection, urinary tract infection), urinary retention, bleeding or hematoma, blood transfusion, ileus or small bowel obstruction, ureteral or bowel injury diagnosed postoperatively, vaginal cuff cellulitis or dehiscence, deep vein thrombosis, pulmonary embolism, cardiac complication, readmission, reoperation, mortality, and other rare complications. In cases where minimally invasive hysterectomy (MIH) was planned and the final surgical approach was abdominal hysterectomy, the case was classified as MIH converted to laparotomy.

### 2.6. Outcomes

The primary outcome was the odds of a composite of any postoperative bleeding complication by use of postoperative ketorolac. Secondary outcomes included the components of the primary outcome, as well as postoperative emergency department visits and readmissions. We hypothesized that the odds of experiencing bleeding complications would not be affected by ketorolac use postoperatively. The American College of Obstetrics and Gynecology’s recommendations for ERAS protocol development discuss multimodal perioperative analgesia, including but not limited to acetaminophen, celecoxib, and gabapentin [19]. We therefore further performed a sub-analysis of patients who received both celecoxib preoperatively and ketorolac postoperatively compared to controls who did not receive either medication.

### 2.7. Statistical Analysis

For categorical variables, we used Chi-square test and Fisher’s exact test as appropriate. For continuous variables, we used Student’s *t*-test. Categorical variables were reported as *n* (%), and continuous variables were reported as the mean (standard deviation). Multivariable logistic regression analysis was performed to identify variables independently associated with the primary outcome. The multivariable logistic regression analysis models included factors that reached a statistically significant difference in the univariate analysis and are clinically relevant. These included, age, American Society of Anesthesiology category, anticoagulant treatment, polytherapy, uterus size, surgical approach, increased surgical complexity, and lysis of adhesions. Results were reported as adjusted odds ratios (aORs) and 95% confidence intervals (CIs). A 2-sided *p*-value < 0.05 was considered statistically significant. Statistical analyses were performed using Software Package for Statistics and Simulation (IBM SPSS version 27, IBM Corp, Armonk, NY, USA), EZR (version 1.55) and R (R Core Team 2021, version 4.1.2; R Foundation for Statistical Computing, Vienna, Austria).

## 3. Results

### 3.1. Patient Characteristics

A total of 4236 hysterectomies for benign indications were performed during the study period and were included in the analysis. Of these, 3236 (76%) patients received ketorolac postoperatively and 1000 (24%) did not (Table 1). Patients receiving ketorolac were younger by 3.5 years (*p* < 0.001). They had a lower BMI and ASA class and were more likely to have commercial insurance (*p* < 0.001). They were less likely to have diabetes mellitus or hypertension (*p* < 0.001).

Uterine fibroids or endometriosis was the most common indication for hysterectomy for those who received ketorolac (*p* < 0.001, Table 2), while pelvic mass, risk reduction, a premalignant condition, and pelvic organ prolapse or incontinence were indications for surgery in those who did not receive ketorolac (*p* < 0.001). Surgical approaches differed significantly between groups. A laparoscopic approach (*p* < 0.001) and supracervical hysterectomy (*p* = 0.001) were more common in the ketorolac group. Patients whose treatment involved endometriosis excision (*p* < 0.001), lysis of adhesions (*p* = 0.006), and increased surgical complexity (*p* < 0.001) received ketorolac more often. The ketorolac group also had an overall lower rate of anticoagulation use, lower EBL, shorter surgery length, shorter hospital stay, and higher rate of same-day discharge compared to the group that did not receive ketorolac (*p* < 0.001 for all).

### 3.2. Postoperative Bleeding Outcomes

The primary and secondary outcomes are presented in Table 3. Postoperative bleeding as a composite occurred less frequently in the ketorolac group (2.1% vs. 4.1%, *p* = 0.001), as did transfusion (1.7% vs. 3.6%, *p* = 0.001). There was no difference between groups in the proportion of reoperation for bleeding, readmission for bleeding, overall emergency department visits, or readmissions. There was no difference between groups for those who had excessive bleeding or intraoperative transfusion (Table 2). There was no significant difference in overall intraoperative complications (*p* = 0.070). Major perioperative complications were less likely to have occurred in patients who received ketorolac (*p* = 0.046). The only postoperative complication that differed significantly between the groups was ileus—those who had not received ketorolac were more likely to develop an ileus (*p* = 0.034).

In multivariable regression analysis, there was no association between ketorolac administration and the risk of composite postoperative bleeding (aOR 1.02, 95% CI 0.36–2.88, Table 4).

### 3.3. Sub-Analysis Outcomes

Among the ketorolac group, 19.9% (*n* = 644) received preoperative celecoxib (Table 2). Our sub-group analysis is shown in Supplementary Table S1. There was less overall postoperative bleeding for the celecoxib-plus-ketorolac group compared to the controls (1.1% vs. 4.1%, *p* < 0.001). Similar to our primary analysis, there were no significant differences in intraoperative complications, while perioperative complications were more common among the non-exposed group (7.5% vs. 13.1%, *p* < 0.001). Multivariable regression analysis revealed no increased risk of composite postoperative bleeding between the exposed and control groups (Supplementary Table S2).

## 4. Discussion

### 4.1. Principal Findings

Among our cohort of patients undergoing hysterectomies for benign indications, there was no association between postoperative ketorolac administration and the risk of postoperative bleeding complications. Reoperations and readmissions for bleeding were comparable between groups, while the risk of postoperative transfusion was decreased for those who had received ketorolac. Perioperative complications overall were also significantly less common in the ketorolac group. There were no differences in intraoperative complications or 30-day postoperative complications except for ileus, which was less frequent in the ketorolac group. Our sub-group analysis of preoperative celecoxib with postoperative ketorolac compared to the control group did not find an additive effect of celecoxib on postoperative bleeding risk.

### 4.2. Results in the Context of What Is Known

NSAIDs, particularly the parenteral option, ketorolac, have previously been shown to decrease opioid use and improve analgesia postoperatively [2,3]. However, concern that ketorolac will increase the risk of bleeding complications after surgery persists. This is largely due to studies showing increased bleeding times associated with ketorolac injections, a characteristic that has not been shown to be a reliable predictor of perioperative bleeding [7,20,21]. In addition, a post-marketing surveillance cohort study of ketorolac, which revealed a significant increase in bleeding with higher doses, older patients, and treatment courses longer than five days, prompted the introduction of a black-box warning against its perioperative use [9,10]. Importantly, the reported definitions of operative site bleeding, the primary outcome, included mention of any bleeding from the surgical site, such as blood spotting—an expected sequela of any procedure. Clinically serious operative site bleeding occurred rarely and not significantly more in the ketorolac-exposed cohort. Since the warning was implemented, many studies have been published supporting the use of ketorolac perioperatively for various procedures [3,16,22]. Two recent meta-analyses showed no significant difference in clinically significant bleeding complications between NSAID or ketorolac use and standard opioid use [11,12]. There are, however, studies examining this risk that demonstrated variation between specific surgical procedures, highlighting the importance of exploring the risk within our own field of gynecology [12,13].

Few published studies have assessed bleeding risks for gynecologic procedures [15,16,23,24]. Balestrieri et al. explored the timing of ketorolac administration in open myomectomy and total abdominal hysterectomy and found improved patient experience, tolerability of pain medications, and ease of nursing care, especially with intraoperative administration [15]. Shah et al. found no difference in hemoglobin level or total use of narcotics after vaginal reconstructive surgery among those who did and did not receive ketorolac [16]. These studies were both limited by their sample size. We performed a literature review to identify studies on the risk of bleeding after hysterectomy with ketorolac administration. To the best of our knowledge, our study is the first to examine bleeding risk associated with ketorolac following hysterectomy. Our findings agree with prior meta-analyses that found no increase in postoperative bleeding complications with ketorolac administration [12].

The composite bleeding complication risk was significantly lower in the ketorolac group in the univariate analysis. One could argue that this is due to provider bias against using ketorolac in cases perceived to have a higher risk of bleeding complications. This hypothesis may be supported by our finding of lower rates of major perioperative complications, lower EBL, higher same-day discharge rates, and younger demographics with fewer co-morbidities in the ketorolac group. However, after adjustment for potential confounders, we found no association with ketorolac use and the composite bleeding outcome. Our data also suggest that there was no difference in rate of ketorolac use in cases with excessive bleeding and intraoperative transfusion.

Notably, patients with cases of higher surgical complexity (i.e., deeply infiltrative endometriosis, extensive lysis of adhesions) were more likely to receive ketorolac. This may reflect an anticipation of increased pain management needs following more complex procedures overriding a concern for bleeding risks. These cases may also lead to high rates of polytherapy, which our analysis adjusts for. Our sub-analysis specifically addresses celecoxib use and reflects no difference in bleeding risks. Additionally, interpretation of our data should include an understanding that our Minimally Invasive Gynecologic Surgery (MIGS) division, which performs a plurality (39.1%) of hysterectomies at our institution, implements ERAS protocols that include ketorolac and celecoxib use [25]. Patients with indications of uterine fibroids and endometriosis and those in whom a laparoscopic approach was used were more likely to receive ketorolac, which is consistent with the MIGS predominance of hysterectomies.

### 4.3. Clinical and Research Implications

As our findings did not show increased risk of postoperative bleeding complications, we support ketorolac administration after hysterectomy for benign indications. Providers should also be reassured that ketorolac may be given in cases with increased surgical complexity and when anticoagulation medication or celecoxib was given perioperatively. Further exploration of the data is necessary to weigh the risk of ketorolac use in those with surgical indications and surgical approaches that may increase a patient’s bleeding risk, such as myomectomy and cancer.

### 4.4. Strengths and Limitations

A strength of this study is that bleeding complication risks have not yet been studied in those undergoing hysterectomy. Importantly, our study includes a large cohort of patients who did and did not receive ketorolac after undergoing a hysterectomy, improving our ability to detect significant differences in bleeding risk and specific outcomes. Additionally, the hysterectomies included were procedures performed by a variety of providers at a large, quaternary care academic institution, adding to the generalizability of the results.

The main limitation of this study is its retrospective study design, which introduces bias, and factors that may not have been assessed, such as postoperative visits at community practices with outside electronic medical records. The single-center design, limited to one quaternary academic hospital, also limits the generalizability of the results. As previously discussed, hysterectomies at our institution are predominantly laparoscopic and MIGS. Additionally, we were not able to explore certain features of ketorolac use, such as the duration of use, timing of administration, and dosing, limiting our ability to comment on these factors, which the prior post-marketing surveillance study raised concerns about [9]. We were also unable to comment on other common safety concerns associated with NSAIDs, including kidney function and gastroenterological effects. However, these concerns can be considered minimal at the doses and duration administered at our institution. In addition, because pain levels were not available in our data, they were not directly assessed as a measure of ketorolac’s efficacy for pain control. Finally, preoperative to postoperative hemoglobin level changes were not evaluated. Postoperative hemoglobin levels were available in only 674 (15.9%) of cases, as blood count is not routinely performed postoperatively at our institution unless clinically indicated. However, this variable has limited clinical utility compared to the primary outcome.

## 5. Conclusions

A multimodal approach to pain management in the postoperative period is a major focus in the context of the opioid epidemic. Our results support the use of ketorolac postoperatively following hysterectomy. The lack of increased risk of bleeding complications with ketorolac use provides further support for broad implementation of ERAS for gynecologic procedures. Our results also suggest that other non-bleeding-related postoperative complications are not increased by ketorolac exposure. Future studies should reevaluate the association between ketorolac exposure and bleeding after hysterectomy and examine other gynecologic procedures with elevated bleeding risks, such as myomectomy, to further inform safe use of ketorolac within the field of gynecology.

## Figures and Tables

**Table 1:** Patient demographic and clinical characteristics, stratified by ketorolac administration.

Characteristic	Ketorolac (*n* = 3236)	No Ketorolac (*n* = 1000)	*p* Value
Age (y)	49.8 (11.1)	53.2 (12.8)	<0.001
BMI (kg/m^2^)	27.1 (6.3)	28.1 (6.8)	<0.001
ASA class			<0.001
1	337 (19.8)	38 (9.2)	
2	1141 (67.0)	291 (70.6)	
3/4	226 (13.3)	83 (20.2)	
Diabetes mellitus	163 (5.0)	91 (9.1)	<0.001
Hypertension	345 (10.7)	182 (18.2)	<0.001
Race			0.222
American Indian or Alaska native	20 (0.6)	3 (0.3)	
Asian	310 (9.6)	116 (11.6)	
Black or African American	493 (15.2)	176 (17.6)	
Native Hawaiian or other Pacific Islander	7 (0.2)	4 (0.4)	
Other or unknown	314 (9.7)	98 (9.8)	
White	2092 (64.6)	603 (60.3)	
Ethnicity			0.836
Hispanic	587 (18.1)	180 (18.0)	
Non-Hispanic	2608 (80.6)	805 (80.5)	
Unknown	41 (1.3)	15 (1.5)	
Insurance type			<0.001
Commercial	2763 (86.1)	767 (77.2)	
Medicare	372 (11.6)	202 (20.3)	
Medi-Cal	47 (1.5)	12 (1.2)	
Charity	12 (0.4)	5 (0.5)	
Other government	14 (0.4)	8 (0.8)	

Data are *n* (%) or mean (standard deviation); BMI, body mass index; ASA, American Society of Anesthesiology.

**Table 2:** Operative characteristics, stratified by ketorolac administration.

Operative Characteristic	Ketorolac (*n* = 3236)	No Ketorolac (*n* = 1000)	*p* Value
Indication for hysterectomy			<0.001
Fibroids	1888 (58.3)	542 (54.2)	
Endometriosis, adenomyosis, or pelvic pain	451 (13.9)	91 (9.1)	
Pelvic mass	173 (5.3)	94 (9.4)	
Abnormal uterine bleeding	251 (7.8)	78 (7.8)	
Gender-affirming	81 (2.5)	22 (2.2)	
Risk-reducing	124 (3.8)	51 (5.1)	
Premalignant conditions	166 (5.1)	68 (6.8)	
Pelvic organs prolapse or stress incontinence	72 (2.2)	34 (3.4)	
Other	30 (0.9)	20 (2.0)	
Celecoxib before surgery	644 (19.9)	-	
Anticoagulant administration	334 (10.3)	147 (14.7)	<0.001
Surgical approach			<0.001
Abdominal	746 (23.1)	259 (25.9)	
Laparoscopic or robotic	2343 (72.4)	665 (66.5)	
Vaginal	147 (4.5)	76 (7.6)	
Robot-assisted surgery	526 (16.3)	158 (15.8)	0.768
Hysterectomy type			0.001
Total	2559 (79.1)	839 (83.9)	
Supracervical	677 (20.9)	161 (16.1)	
Uterus size, cm	12.5 (5.0)	11.6 (4.7)	<0.001
Mini laparotomy	16 (0.5)	8 (0.8)	0.332
Unplanned conversion to laparotomy ^a^	71 (2.9)	24 (3.2)	0.584
Concomitant procedures			
Lysis of adhesions	426 (13.2)	167 (16.7)	0.006
Endometriosis excision	305 (9.4)	36 (3.6)	<0.001
Ureterolysis	250 (7.7)	67 (6.7)	0.303
Appendectomy	57 (1.8)	15 (1.5)	0.675
Increased surgical complexity	153 (4.7)	17 (1.7)	<0.001
Excessive intraoperative bleeding	12 (0.4)	9 (0.9)	0.066
Intraoperative transfusion	34 (1.1)	16 (1.6)	0.179
Estimated blood loss (mL)	134.8 (228.)	192.2 (322.2)	<0.001
Total surgery time (minutes)	140.8 (68.6)	154.4 (96.9)	<0.001
Total days of hospitalization	1.30 (1.59)	1.99 (3.50)	<0.001
Same-day discharge	1516 (46.8)	401 (40.1)	<0.001

Data are shown as *n* (%) or mean (standard deviation). ^a^ The denominator excludes cases that were scheduled as abdominal hysterectomy (i.e., were not converted following diagnostic laparoscopy).

**Table 3:** Perioperative outcomes, stratified by ketorolac administration.

	Ketorolac (*n* = 3236)	No Ketorolac (*n* = 1000)	*p* Value
Bleeding outcomes			
Composite postoperative bleeding ^a^	68 (2.1)	41 (4.1)	0.001
Postoperative transfusion	54 (1.7)	36 (3.6)	0.001
Reoperation for bleeding	10 (0.3)	3 (0.3)	>0.999
Readmission for bleeding	12 (0.4)	4 (0.4)	>0.999
Other perioperative outcomes			
Any perioperative complication	356 (11.0)	131 (13.1)	0.070
Major perioperative complication	70 (2.2)	33 (3.3)	0.046
Minor perioperative complication	318 (9.8)	111 (11.1)	0.255
Intraoperative complications			
Any complications	34 (1.1)	18 (1.8)	0.070
Bowel injury	4 (0.1)	7 (0.7)	0.005
Cystotomy	16 (0.5)	3 (0.3)	0.591
Postoperative complications			
Emergency department visit	148 (4.6)	33 (3.3)	0.089
Readmission	42 (1.3)	7 (0.7)	0.131
Vaginal bleeding	24 (0.7)	7 (0.7)	>0.999
Urinary retention	43 (1.3)	8 (0.8)	0.244
Urinary tract infection	85 (2.6)	20 (2.0)	0.296
Ileus	33 (1.0)	19 (1.9)	0.034
Small bowel obstruction	20 (0.6)	10 (1.0)	0.201
Cellulitis	2 (0.1)	2 (0.2)	0.238
Other infection	32 (1.0)	7 (0.7)	0.569
Vaginal cuff dehiscence	13 (0.4)	3 (0.3)	0.776
Cutaneous wound complication	25 (0.8)	10 (1.0)	0.548
Pelvic abscess	25 (0.8)	14 (1.4)	0.086
Deep vein thrombosis	7 (0.2)	3 (0.3)	0.709
Reoperation	29 (0.9)	8 (0.8)	>0.999

Data are *n* (%); ^a^ Any readmission for bleeding, postoperative transfusion, or reoperation for bleeding.

**Table 4:** Multivariable regression analysis of factors associated with composite postoperative bleeding ^a^.

	Adjusted Odds Ratio	95% Confidence Interval
Ketorolac administration	1.03	(0.36–2.93)

Adjusted for age, American Society of Anesthesiology category, celecoxib before surgery, anticoagulant treatment, uterus size, surgical approach, increased surgical complexity, and lysis of adhesions. ^a^ Any readmission for bleeding, postoperative transfusion, or reoperation for bleeding.

## Data Availability

The datasets presented in this article are not readily available for patients protection.

## Supplementary Materials

The following supporting information can be downloaded at https://www.mdpi.com/article/10.3390/jcm15020869/s1, Supplementary Table S1: Perioperative Outcomes, stratified by pre-operative celecoxib administration in addition to ketorolac administration. Supplementary Table S2: Multivariable regression analysis of factors associated with composite postoperative bleeding among patients who received celecoxib pre-operatively and ketorolac postoperatively versus controls.
