# Supplementary material for: Routine Ketorolac Use for Postoperative Pain Does Not Increase Bleeding Risk After Hysterectomy
Source: J Clin Med. 2026 Jan 21;15(2):869. doi: 10.3390/jcm15020869 (PMC12842528; doi:10.3390/jcm15020869)
Supplement: Supplementary file 1 [file jcm-15-00869-s001.zip › jcm-4101111-supplementary.pdf]

Supplementary Table S1. Perioperative Outcomes, stratified by pre-operative celecoxib administration in addition to ketorolac administration.

	Celecoxib (n=644)	No celecoxib (n=1000)	p Value
**Bleeding outcomes**			
Composite postoperative bleeding*	7 (1.1)	41 (4.1)	<.001
Postoperative transfusion	5 (0.8)	36 (3.6)	<.001
Reoperation for bleeding	1 (0.2)	3 (0.3)	1
Readmission for bleeding	1 (0.2)	4 (0.4)	.654
**Other perioperative outcomes**			
Any perioperative complication	48 (7.5)	131 (13.1)	<.001
Major perioperative complication	11 (1.7)	33 (3.3)	.060
Minor perioperative complication	41 (6.4)	111 (11.1)	.001
**Intraoperative complications**			
Any complications	8 (1.2)	18 (1.8)	.424
Bowel injury	1 (0.2)	7 (0.7)	.159
Cystotomy	5 (0.8)	3 (0.3)	.275
**Postoperative complications**			
Emergency department visit	24 (3.7)	33 (3.3)	.680
Readmission	6 (0.9)	7 (0.7)	.584
Vaginal bleeding	3 (0.5)	7 (0.7)	.445
Urinary retention	8 (1.2)	8 (0.8)	.443
Urinary tract infection	9 (1.4)	20 (2.0)	.445
Ileus	1 (0.2)	17 (1.7)	.003
Small bowel obstruction	3 (0.5)	10 (1.0)	.270
Cellulitis	1 (0.2)	2 (0.2)	
Other infection	2 (0.3)	7 (0.7)	.496
Vaginal cuff dehiscence	3 (0.5)	3 (0.3)	.684
Cutaneous wound complication	3 (0.5)	10 (1.0)	.270
Pelvic abscess	4 (0.6)	14 (1.4)	.154
Deep vein thrombosis	0 (0.0	3 (0.3)	.684
Reoperation	4 (0.6)	8 (0.8)	.774

Data are n (%)

* Any readmission for bleeding, postoperative transfusion or reoperation for bleeding

Supplementary Table S2. Multivariable regression analysis of factors associated with composite postoperative bleeding* among patients who received celecoxib pre-operatively and ketorolac post-operatively versus controls

	Adjusted odds ratio	95% Confidence Interval
Celecoxib and ketorolac administration	0.95	0.31-2.90

Adjusted for: American Society of Anesthesiology category, anticoagulants treatment, surgical approach, increased surgical complexity, endometriosis excision.
* Any readmission for bleeding, postoperative transfusion or reoperation for bleeding
